# Effect of anesthesia administration method on apgar scores of infants born to women undergoing elective cesarean section

**DOI:** 10.1186/s12871-023-02098-w

**Published:** 2023-04-27

**Authors:** Chipo Gwanzura, Samuel Gavi, Marcia Mangiza, Faith Vhenekai Moyo, Matthew C. Lohman, Taazadza Nhemachena, Tsungai Chipato

**Affiliations:** 1 Department of Obstetrics and Gynecology, Faculty of Medicine and Health Science, University of Zimbabwe, Harare, Zimbabwe; 2 Department of Epidemiology and Biostatistics, Arnold School of Public Health, University of South Carolina, 915 Greene Street, Columbia, SC 29208 USA; 3 Department of Pediatrics, Faculty of Medicine and Health Science, University of Zimbabwe, Harare, Zimbabwe; 4 Department of Anesthesia and Critical Care Medicine, Sally Mugabe Central Hospital, PO Box ST14 Southerton, Harare, Zimbabwe; 5 Clinical Trials Research Centre, College of Health Sciences, University of Zimbabwe, Harare, Zimbabwe

**Keywords:** Apgar scores, Spinal anesthesia, General anesthesia, Elective cesarean section, Marginal structural logistic modelling

## Abstract

**Background:**

Neonatal health at delivery as measured by apgar scores is an important outcome. This study was done to assess the impact of anesthesia on Apgar 1-minute and 5-minute scores of infants delivered through elective cesarean section in Zimbabwe.

**Methods:**

We carried out a secondary analysis of data from the Efficacy of Tranexamic Acid in Preventing Postpartum Hemorrhage (ETAPPH) clinical trial in Zimbabwe. Outcomes measured were infant Apgar scores at 1 and 5 min, exposure was the administration of either a general (intravenous propofol/ketamine/sodium thiopental) or spinal (hyperbaric bupivacaine 0.5%) anesthesia for anesthesia during the elective cesarean section procedure. Marginal Structural Logistic Modelling (MSM) using an unstabilized Inverse Probability Treatment Weight (IPTW) estimator was used to assess the relationship between anesthetic administration method and infant Apgar scores.

**Results:**

Four hundred and twenty-one (421) women who had an elective caesarean section in the ETAPPH study had their infants assessed for Apgar scores. Comparing general anesthesia to spinal anesthesia, spinal anesthesia was related to good Apgar scores at 1-minute (adjusted odds ratio [aOR] = 4.0, 95% Confidence Interval = 1.5–10.7, sensitivity analysis E-value = 3.41). Spinal anesthetic administration was also related to good Apgar scores at 5 min (adjusted odds ratio [aOR] = 6.2, 95% Confidence Interval = 1.6–23.1, sensitivity analysis E-value = 4.42).

**Conclusions:**

When providing anesthesia for patients undergoing elective cesarean section, care should be taken on the method of administration of anesthetic agents. General anesthesia tends to depress Apgar scores at 1 min, although most neonates recover and have better scores at 5 min. Spinal anesthesia should be the first choice whenever possible.

**Trial registration:**

The clinical trial from which data of this study was abstracted was registered under clinical trials registration number NCT04733157.

## Background

Global estimates show neonatal mortality has significantly declined by more than 50% worldwide between 1990 and 2017, from 64.5 infants per 1,000 live births in 1990 to 28.2 infants per 1,000 live births in 2019 [1, 2]. The sustainable development goals (SDGs) on health have targeted 2030 as a time point when neonatal mortality should have declined to 12 per 1,000 live births [3, 4]. While these are significant reductions and modest goals, the Sub-Saharan African region still experiences higher than average neonatal mortality and morbidity. Other low-middle income countries have been experiencing the same trends of lower neonatal mortality and morbidity even though they remain higher than high income countries [5]. Safe obstetric practices, access to high-quality antenatal and postnatal care, and strong health systems have been the major drivers of observed reductions in neonatal mortality and morbidity [6]. General causes of neonatal mortality include low birth weight, premature birth, birth asphyxia, and infections (sepsis/pneumonia, tetanus, diarrhea), which can be prevented by improving access to skilled health professionals for antenatal, birth, and postnatal care among others [7].

Cesarean section when available is a good intervention for better neonatal health outcomes. An important indicator of neonatal health is the Apgar 1 and 5 scores. Apgar scores at 1-minute show how well the baby is doing after the birth process while Apgar score at 5-minutes can be a predictor of health and mortality in the infant [8]. Determinants of Apgar scores include preterm birth, type of anesthesia, and stress of mother during delivery either by vaginal, elective or emergency cesarean Sects. [9, 10]. In other settings, HIV infection with malaria co-infection was found to result in lower apgar scores at five minutes [11]. Assessing Apgar score is essential to understand the impact of these on child health outcomes and thus promote safe obstetric practices that improve maternal and newborn health. To estimate the potential risk for mortality in newborn children, birth weight and Apgar scores have been used as proxy indicators of outcomes later in life that include mortality and developmental problems [12, 13]. The choice of anesthesia used during delivery has been shown to affect apgar scores. A study by Ratcliffe showed that there was a greater percentage of neonates who had an apgar score greater than 7 when their mothers had been given spinal anesthesia compared to general anesthesia [9]. In contrast a few studies have shown that anesthesia technique did not affect apgar scores. A prospective randomized study by Mancuso showed that use of general or spinal anesthesia did not result in significant differences in apgar outcomes in infants though spinal anesthesia showed better effect on neonatal outcomes [14]. Similar observations had been made by Ebrle et al. which showed that anesthethia type did not alter apgar scores in infants if we control for infant’s birth weight. [10]. Sendag et al. also had similar conclusions that apgar scores did not differ by type of anesthesia used [15]. A secondary data analysis by Harazim et al. showed that there was no difference in apgar scores by anesthesia method [16].

**Fig. 1:**
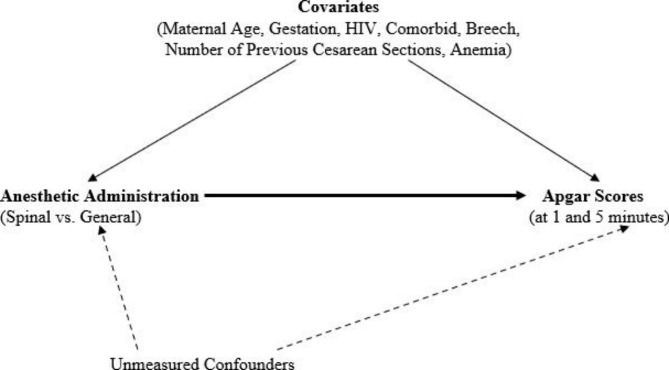
A directed acyclic graph describing the relationship between Spinal/General anesthetic administration and Apgar 1-minute and 5-minute scores

The objective of this analysis was to assess the relationship between the type of anesthesia administered during delivery (spinal vs. general) and neonatal Apgar 1- and 5-minute scores using MSM analysis [17, 18]. MSM has the unique advantage of controlling time-varying confounders, unmeasured confounders, and selection bias through Inverse Proportional Treatment Weighting (IPTW) estimator calculation. This is a better approach compared to traditional methods of logistic regression analysis and allows us to make a stronger interpretation of the relationship between anesthesia and apgar scores; most studies investigating this had not used a causal interpretation approach. Another advantage of our approach in comparison to other studies that have analysed secondary data but not using strong analytical approaches is that these techniques are not affected by small sample sizes which would reduce the strength of observations. The study hypothesizes that the use of general anesthesia during delivery is associated with adverse Apgar scores at 1 and 5 min (Fig. 1).

## Methods

We carried out a secondary analysis of data from a clinical trial designed to study the efficacy of tranexamic acid in preventing postpartum hemorrhage (ETAPPH) after elective cesarean section. This analysis assessed the relationship between method of anesthetic delivery method (general or spinal) for maternal anesthesia on neonatal Apgar scores at 1 and 5 min.

### Study setting

The ETAPPH trial was conducted at two University of Zimbabwe affiliated hospitals which serve as referral centres; Sally Mugabe Central Hospital (SMCH) and Parirenyatwa Group of Hospitals (Mbuya Nehanda Maternity Hospital-MNMH). On average, 60 elective caesarean sections are done at SMCH per month, while MNMH does 80 elective caesarean section deliveries per month. Care of mothers during delivery was done by senior resident medical officers (SRMO’s), senior hospital officers (SHOs), and registrars or consultants. Anesthetic delivery was conducted by specialist anesthesiologists, registrars, SHOs, nurses and student nurse anesthesiologists (SNA’s) depending on the patient requirements as decided by the team.

### Participants

Study participants were women undergoing elective cesarean sections at SMCH and Parirenyatwa Hospitals. Inclusion criteria were ability to provide signed informed consent, estimated gestational age of 38 weeks or more, requiring elective cesarean section (defined as caesarean section performed before onset of labour), and with a live intrauterine fetus. If a minor met the inclusion criteria informed consent was sought from legally authorized representatives such as the parent or guardian. Potential participants were excluded from the study if they had placental abruption, emergency caesarean section, history of epilepsy or seizures, sickle cell disease, intrauterine fetal demise, eclampsia/HELLP syndrome, administration of anticoagulants or antiplatelet agents in the week before delivery.

### Exposure

The predictor variable was type of anesthesia given to the mother during delivery spinal (hyperbaric bupivacaine (7.5 mg) 0.5%) or general anesthesia (ketamine (1-4.5 g/kg)/propofol (2-2.5 mg/kg)/sodium thiopental (3–4 mg/kg)). Indication for general or spinal anesthesia for elective surgery were dependent on patient preference, availability of drugs and equipment like spinal needles and hyperbaric bupivacaine, and choice of attending practitioner. All patients volunteered to participate in the study; those with emergency conditions such as fetal distress or other emergent indications were excluded.

### Covariates

Other covariates which are potential confounders included age of participant, comorbidities, number of previous cesarean sections, gestational age in weeks, maternal anemia, Human Immunodeficiency Virus (HIV) infection, and breech presentation. (Fig. 1) is a directed acyclic graph showing a conceptual causal path for the relationship between method of anesthetic delivery and infant apgar scores at 1 and 5 min. No patient identifiers were abstracted or used to do the analysis.

### Outcome

Neonatal outcomes that were measured in the analysis were Apgar scores at 1 and 5 min on the day of delivery. Apgar scores are measured from 1 to 10, the scores were categorized into two groups for both Apgar scores at 1 and 5 min. Apgar scores from 1 to 7 (coded 0) and above 8–10 (coded 1) for Apgar scores at 1 min, and Apgar scores from 1 to 7 (coded 0) and 8–10 (coded 1) for Apgar scores at 5 min. This categorization of apgar scores was adapted by the consensus of the American Academy of Pediatrics on measurement of apgar scores and their usefulness [19].

### Statistical methods

**Fig. 2:**
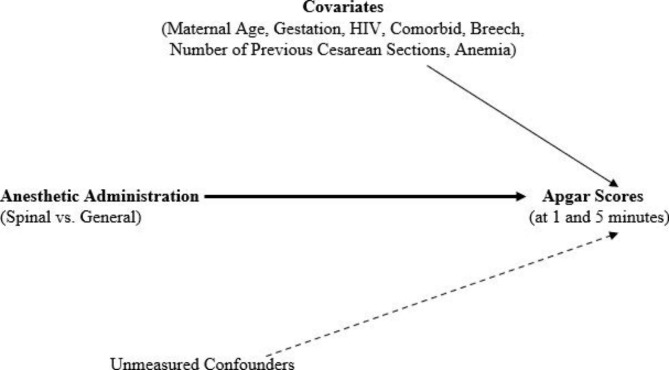
A directed acyclic graph describing the relationship between Spinal/General anesthetic administration and Apgar 1-minute and 5-minute scores after Inverse Probability Treatment Weighting

We carried out an analysis of the relationship between anesthetic administration method and Apgar 1 and 5 scores using marginal structural modelling [17, 18]. Unstabilized inverse probability treatment weighting estimation on the exposure was done using the variables age, breech presentation, number of cesarean sections, gestational age, comorbidities, HIV infection, and anemia; the weights were used in marginal structural model analysis. Sensitivity analysis without assumptions was performed to evaluate impact of unmeasured confounders reporting the E-value [20, 21]. To cover for selection bias due to missing data on outcomes, we created an unstabilized weight for missing values which we then multiplied with the unstabilized weight from treatment, to create a new unstabilized weight for the pseudo population created by IPTW. This was used in the MSM logistic regression analysis of apgar scores and anesthesia administration. After IPTW, the covariates in the pseudo population were expected to be no longer associated with anesthesia (spinal or general) as illustrated in (Fig. 2). The data analysis for this paper was generated using SAS software, Version [9.4] of the SAS System for Windows.

## Results

**Fig. 3:**
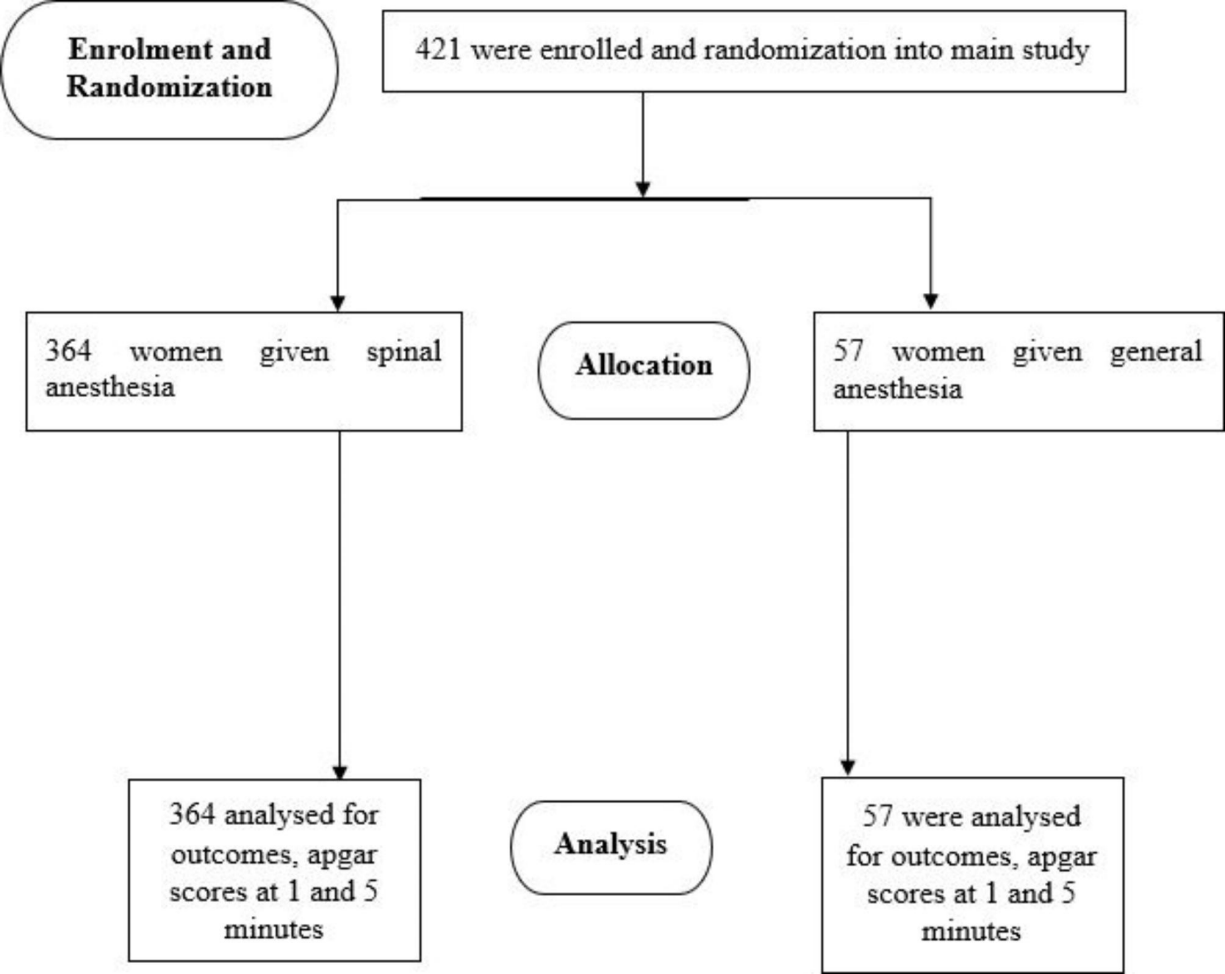
Participant flow diagram

The ETAPPH study enrolled 421 women, 364 women had spinal anesthesia and 57 had general anesthesia during elective cesarean Sect. (10 records were not included as data on apgar scores at 1 and five minutes was missing). Figure 3 is a flow diagram of participant enrollment into the main study. The choice of anesthetic agent to use was based on patient’s choice and in some cases availability of specific agent, with spinal anesthesia being the most frequent choice among anesthesiologists. The distribution of age, weight, and gestation in the two groups was similar. Thirty-seven (37) women who had spinal anesthesia also had a breech delivery, compared to 8 women who had general anesthesia (Table 1).

**Table 1:** Characteristics of Mothers Having Elective Cesarean Section

Variable	General Anesthesia N = 57 (13.5%)	Spinal Anesthesia N = 364 (86.5%)	p-value
Age Less than or equal to 23 years 24–34 years 35 + years	7 (12.3%) 36 (63.1%) 14 (24.6%)	68 (18.7%) 210 (57.7%) 86 (23.6%)	0.5
Anemia Not anemic Anemic	33 (97.1%) 1 (2.9%)	285 (95.6%) 13 (4.4%)	0.4 (Fischer’s exact)
Breech No Breech Presentation Breech Presentation	49 (86.0%) 8 (14.0%)	331 (90.9%) 33 (9.1%)	0.2
Previous Cesarean Sections None One C-Section Two-Five C-Sections	26 (45.6%) 18 (31.6%) 13 (22.8%)	143 (39.3%) 137 (37.6%) 84 (23.1%)	0.6
Comorbid Condition No Comorbid condition At least one other condition	53 (94.7%) 3 (5.3%)	342 (94.5%) 20 (5.5%)	0.9
Gestation 38 weeks 39 weeks >= 40 weeks	13 (22.8%) 19 (33.3%) 25 (43.9%)	149 (40.9%) 103 (28.3%) 112 (30.8%)	0.3
HIV Status Negative Positive	49 (87.5%) 7 (12.5%)	297 (85.1%) 52 (14.9%)	0.6

Table 1 shows that no covariate was associated with a type of anesthesia administered to a participant. Table 2 shows the covariate distribution by method of anesthesia after unstabilized IPTW estimator calculation, IPTW removed residual confounding that may have been due to the covariates as they remained unassociated to exposure of either spinal or general anesthesia, except for gestation.

**Table 2:** Covariate Distribution by Anesthesia Administration after IPTW

Variable	General Anesthesia N = 328 (50.8%)	Spinal Anesthesia N = 318 (49.2%)	p-value
Age Less than or equal to 23 years 24–34 years 35 + years	52 (16.0%) 196 (59.7%) 80 (24.3%)	59 (18.5%) 185 (58.3%) 74 (23.2%)	0.7
Anemia Not anemic anemic	319 (97.2%) 9 (2.8%)	304 (95.6%) 14 (4.4%)	0.3
Breech No Breech Presentation Breech Presentation	293 (89.5%) 35 (10.5%)	284 (89.2%) 34 (10.8%)	0.9
Previous Cesarean Sections None 1 C-Section 2 to 5 C-Sections	149 (45.4%) 115 (35.2%) 64 (19.4%)	128 (40.3%) 115 (36.2%) 75 (23.5%)	0.3
Comorbid Condition No Comorbid condition At least one other condition	322 (98.3%) 6 (1.7%)	307 (96.6%) 11 (3.4%)	0.2
Gestation 38 weeks 39 weeks >= 40 weeks	167 (50.9%) 82 (25.1%) 79 (24.0%)	129 (40.6%) 91 (28.6%) 98 (30.8%)	0.03
HIV Status Negative Positive	284 (86.6%) 44 (13.4%)	275 (86.4%) 43 (13.6%)	0.9

We carried out a MSM analysis to assess the relationship between infant apgar scores at five (1) and five (5) minutes using adjusted odds ratio (**aOR**) as the point estimate. We used a saturated marginal structural logistic model since the exposure (anesthetic method) was dichotomous. Univariate analysis of the unstabilized weight showed a mean value of 2.06, indicating suitability of the variables used in creation of pseudo population by inverse probability treatment weighting. Since IPTW did not completely remove confounding due to gestational age, we controlled for gestational age in the regression model. In the marginal structural model analysis, administration of a spinal anesthetic agent was strongly related with higher Apgar scores (8–10) at 1 min (**aOR** = 4.0, **95% CI** = 1.5 to 10.7, sensitivity analysis E-value = 3.41) compared to administration of general anesthesia. Spinal anesthesia was strongly related to higher Apgar scores (8–10) at 5 min (**aOR** = 6.2, **95% CI** = 1.6 to 23.1, sensitivity analysis E-value = 4.42) compared to administration of general anesthesia [21, 22] (Table 3).

**Table 3:** Adjusted Estimates After a Marginal Structural Logistic Model Analysis

Variable	Apgar score at 1-minute aOR, 95% CI, p-value	Apgar score at 5-minutes aOR, 95% CI, p-value
Anesthetic Administration General (baseline) vs. Spinal Anesthesia	aOR = 4.0, 95% CI = 1.5 to 10.7, p-value = 0.006	aOR = 6.2, 95% CI = 1.6 to 23.1, p-value = 0.007
Gestation	aOR = 1.23 95% CI = 0.6 to 2.7 p-value = 0.4	aOR = 0.7 95% CI = 0.2 to 1.8 p-value = 0.4

## Discussion

The analysis showed a strong causal relationship between method of anesthetic administration (spinal or general) and apgar scores at 1 and 5 min. Higher apgar scores were observed with maternal administration of anesthetic by spinal injection compared to general method. These results are consistent with other studies that have shown that general anesthesia tends to depress apgar scores at 1 min; at apgar 5 many infants will have recovered but the initial depression is theoretically linked to early childhood cognitive issues [9, 10, 14, 15]. Ratcliffe et al. used apgar score cut-off of 7 or greater to show good apgar scores, in our study we used updated guidelines from the American Academy of Pediatrics of a score greater than or equal to 8 to be considered good apgar scores. Sendag et al. used a lower cut off point of 4 for apgar scores to reflect severe depression of apgar scores [15]. As a result, their observations differed from our study where we used a higher cut-off.

We calculated the E value to assess sensitivity of the odds ratio estimates to bias from unmeasured confounders. The E-value represents the strength of the confounder needed to nullify the observed relationship between type of anesthetic drug given and infant apgar scores. Our results showed that a relatively strong unmeasured confounder could alter the observed causal relationship between anesthetic administration method and Apgar scores at 1 and 5 min; compared to the observed effect of spinal or general anesthesia. Confounding by indication may be an unmeasured confounder as doctors with more specialized training and experience see more complicated cases that may require administration of general anesthesia.

The analysis was limited to a subset of data from the ETAPPH trial, and this limited the number of potential confounders or effect modifiers that we could assess which could impact apgar scores. The results of this analysis are generalizable to scenarios where women are given anesthetics for pain management during elective cesarean delivery. Spinal anesthesia is a safer option especially in emergencies where a neonate is potentially exposed to more stress during delivery.

## Data Availability

Data is available upon request from the University of Zimbabwe College of Health Sciences in line with their IT and Research Policies. Data can be requested through Dr Chipo Gwanzura (chipo.gwanzura@gmail.com).

## Abbreviations

SRMO: Senior Resident Medical Officer
SHO: Senior House Officer
JNR: Junior
SNR: Senior
SNA: Student Nurse Anesthesiologist
NA: Nurse Anesthesiologist
ETAPPH: Efficacy of Tranexamic Acid for Post-partum Hemorrhage
